# Tectonic setting for Tsunemori Formation in the Permian accretionary complex of the Akiyoshi Belt, Southwest Japan

**DOI:** 10.1016/j.heliyon.2018.e01084

**Published:** 2018-12-24

**Authors:** Koji Wakita, Ruri Yoshida, Yuki Fushimi

**Affiliations:** Yamaguchi University, Yamaguchi City, Yamaguchi Prefecture, 753-8512, Japan

**Keywords:** Geology, Earth sciences

## Abstract

The purpose of this study is to reexamine the age, depositional environment, and tectonic setting of the Tsunemori Formation. This study provides a new tectonic model on the formation of Tsunemori Formation, which is a key formation to understand the Late Permian subduction and accretionary processes in Japan. The sedimentation age of the Tsunemori Formation is late Middle Permian to early Late Permian, based on radiolarians, such as *Follicucullus* cf. *scholasticus* Ormiston and Babcock reported in this study. This paper disproves the previous theory of atoll carbonates collapse at the trench for the Akiyoshi Limestone. Most of sedimentary rocks of the Tsunemori Formation is not pervasively sheared, are less deformed compared with sedimentary rocks of the typical accretionary complexes. There is no layer parallel or sub-parallel thrusting in the turbidite sequences of the Tsunemori Formation. Mudstone of turbidite and pebbly mudstone is lack of fissility and scaly cleavages. The styles of deformation, occurrence of reworked fossils, presence of calcarenite and limestone breccia suggest that the Tsunemori Formation was not a part of accretionary wedges, but possibly was fore arc or slope basin deposits. Presence of reworked fossils, calcarenite, limestone breccia suggest that a part of the provenance of Tsunemori Formation are limestone exposed at the arc trench gap, which was moved upward to reach the arc trench gap where the Akiyoshi Limestone provides its fragments and blocks into forearc and/or slope basins. Therefore, Tsunemori Formation is a deposit in the forearc basin and/or slope basin rather than trench deposits.

## 1 Introduction

**Fig. 1:**
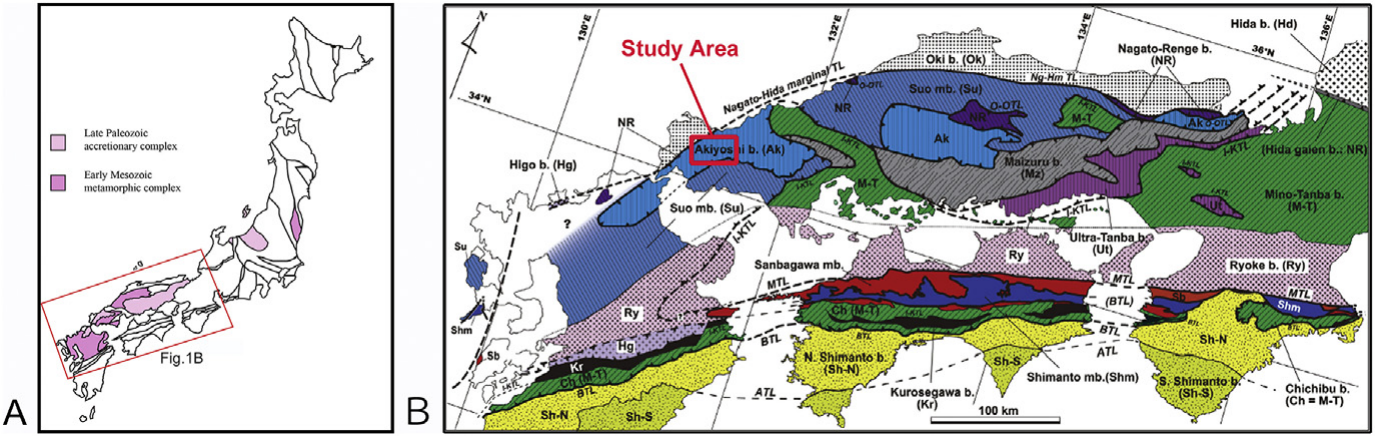
Distribution of late Paleozoic accretionary complex in Japan, and tectonic division in Southwest Japan. A: Distribution of Late Paleozoic accretionary complex and Early Mesozoic metamorphic complex. B: Tectonic division in Southwest Japan (after Isozaki et al., 2010), showing the location of the study area in Yamaguchi Prefecture, Southwest Japan.

The main purpose of this paper is to propose alternative model for the tectonic setting of Tsunemori Formation, which is a key unit to understand the Permian subduction and accretion processes in Japan. Tsunemori Formation is one of major components of the Permian accretionary complex of the Akiyoshi Belt, Southwest Japan (Fig. 1). The Tsunemori Formation is intimately associated with “Akiyoshi Limestone” which was formed as atoll carbonates sitting on volcanic island, which was born in the Panthalassan Ocean (Kanmera and Nishi, 1983; Sano and Kanmera, 1988). The atoll carbonates started to grow up in the Early Carboniferous, and was believed to be growing up until middle Permian time, and accreted at trench in Middle to Late Permian time (Sano and Kanmera, 1988). The Tsunemori Formation is the main target of Sano and Kanmera (1991a, b, c, d) to establish their limestone collapse and accretion model of the Akiyoshi Limestone as well as internal deformation structures, and explained that Tsunemori Formation was a trench deposit, in which Akiyoshi Limestone was accreted as an exotic block.

There are two types of convergent margin, i.e. accretion margin and tectonic erosion margin (e.g., Clift and Vannucchi, 2004). The example of seamount collapse at the trench is observed only at the tectonic erosion margin (e.g., Lallemand et al., 1989). On the other hand, most of seamounts are subducted into the deeper part of the accretionary prism or into mantle without deformation in the accretion margin (Yamazaki and Okamura, 1989). Seamount accretion occurred in the special case of ocean ridge subduction (Vannucchi et al., 2006). Atoll carbonate collapse and accretion do not occur at the same time and at the same convergent margin in present. Thus, the possibility of atoll carbonate collapse and accretion in the convergent margin is controversial. Therefore, we restudied Tsunemori Formation in various aspects such as the distribution, lithology, structures, deformation, and fossil ages. Based on these studies, we discussed the tectonic setting and processes of formation of sedimentary rocks of the Tsunemori Formation, and relationship between Tsunemori Formation and Akiyoshi Limestone.

## 2 Geological setting

### 2.1 Tsunemori Formation

**Fig. 2:**
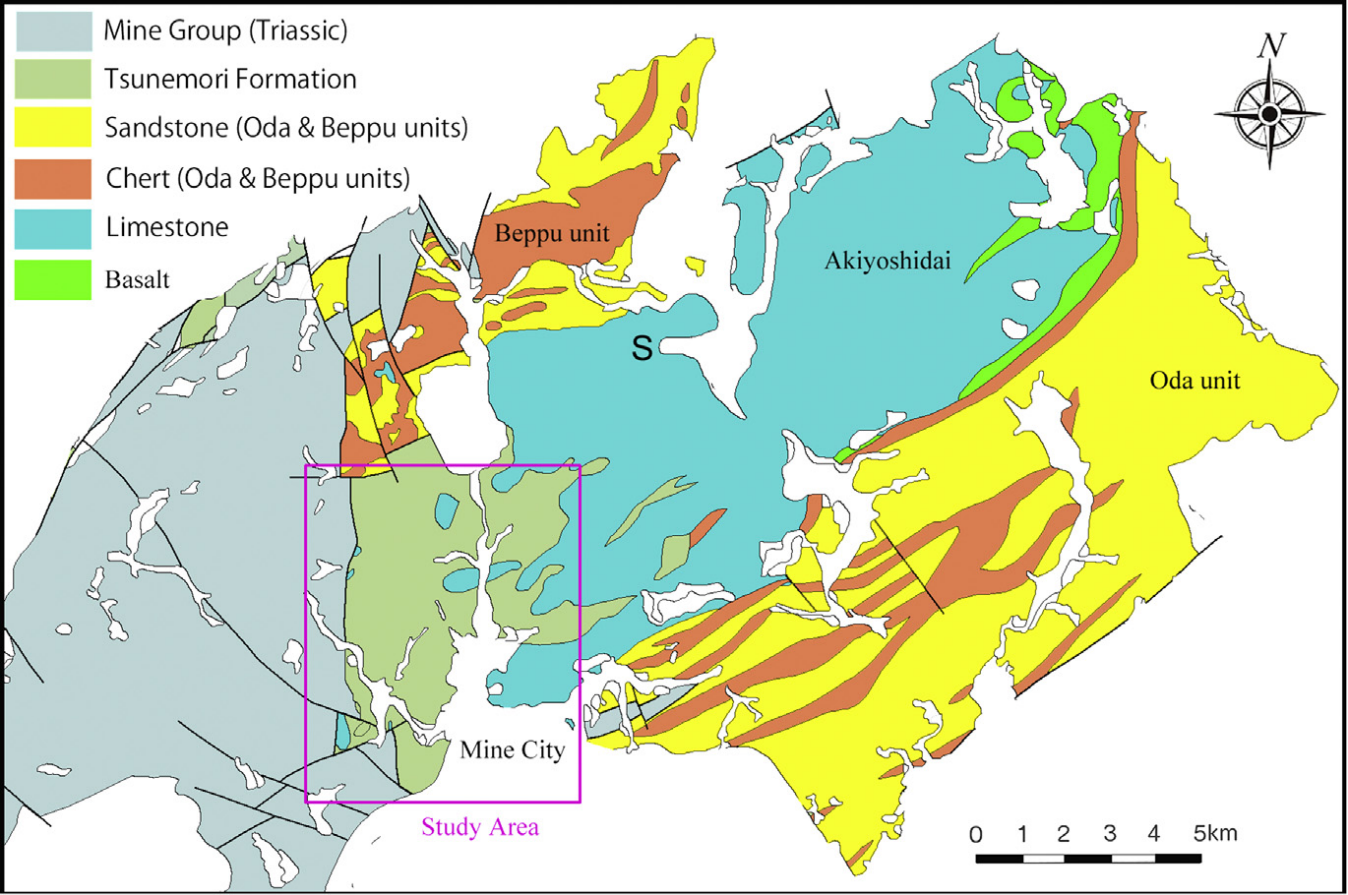
Geology of Akiyoshi area, Yamaguchi Prefecture, Japan. Permian Tsunemori Formation is distributed in the west of the Akiyoshi Limestone and the Oda unit, and on the south of the Beppu unit. Tsunemori Formation is unconformablly overlain by Triassic Mine Group which is distributed in the west of the Tsunemori Formation.

**Fig. 3:**
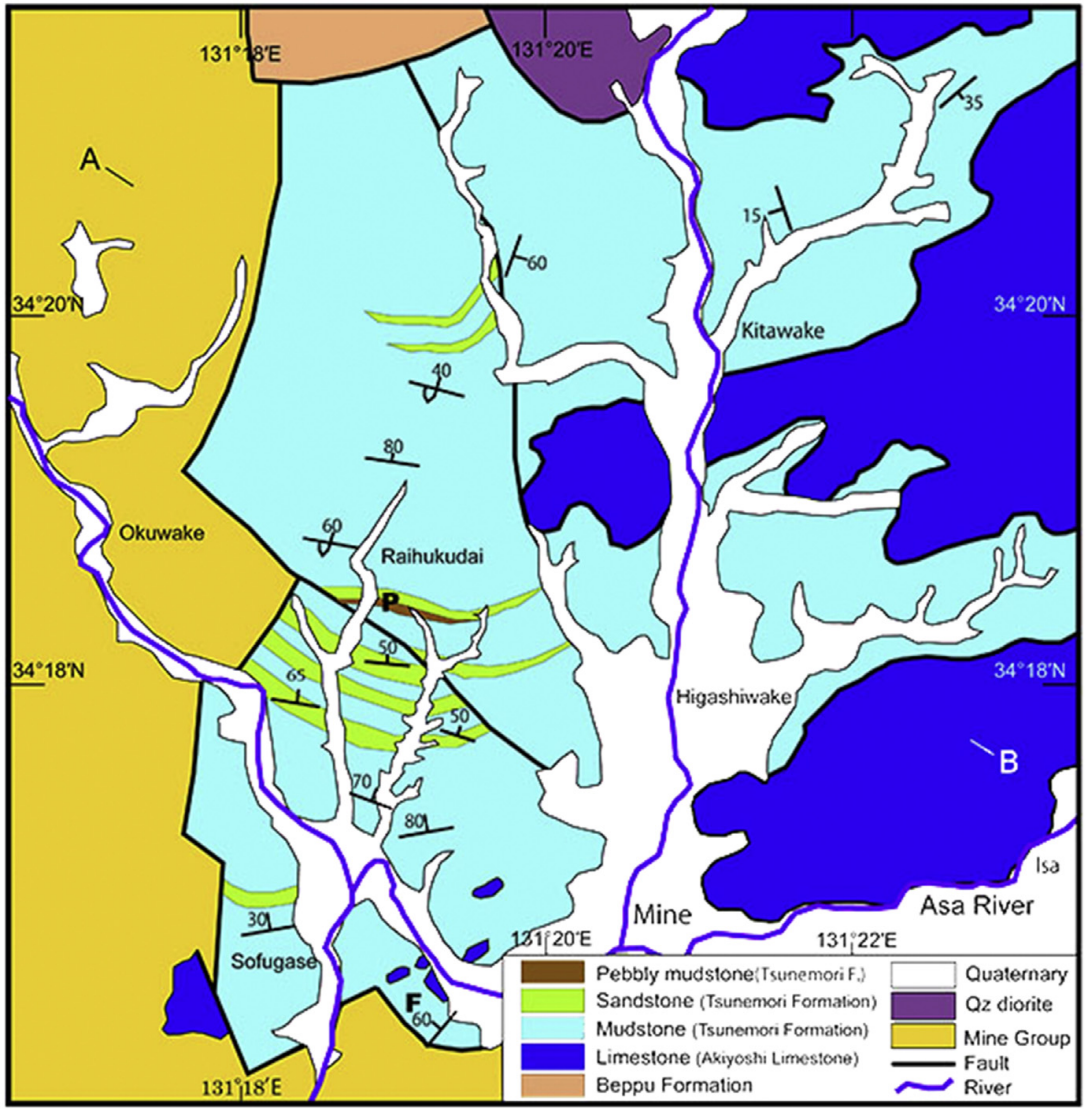
Geologic Map of the Tsunemori Formation. Tsunemori Formation is distributed between Triassic Mine Group to the west and Permian Akiyoshi Limestone to the east. “P” shows the locality of pebbly mudstone showing in Fig. 7. “F” indicates the locality of occurrence of *Follicucullus* cf. *schorasticus* shown in Fig. 8. “T” is the locality of undeformed turbidite of Fig. 6E, F.

**Fig. 4:**
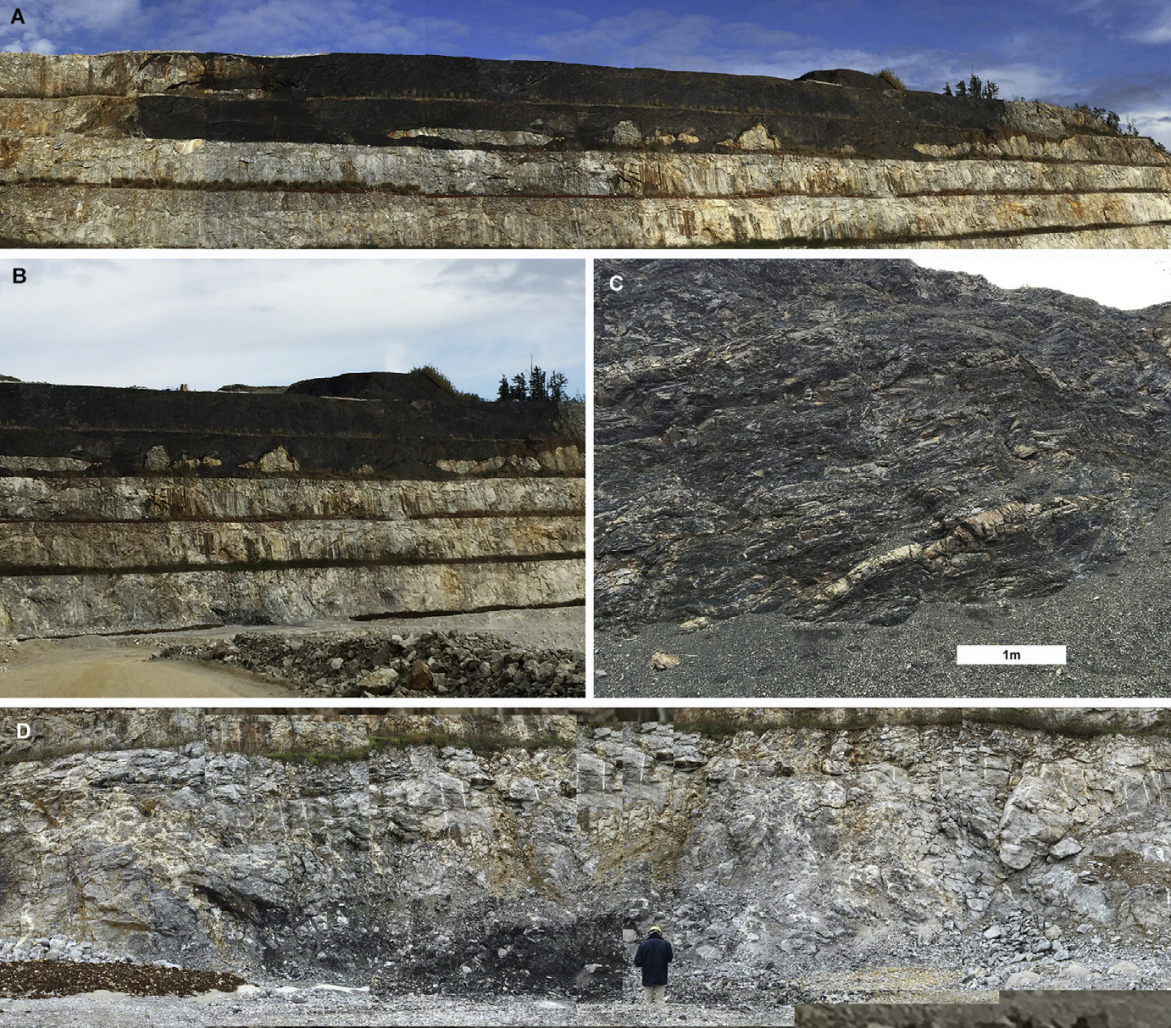
Outcrop of the Akiyoshi Limestone and adjacent Tsunemori Formation at Shuho Mine, Mine City. Locality of the Shuho Mine is shown as “S” in Fig. 2. A: Tsunemori Formation covers the Akiyoshi Limestone on the top (one step is 15m high). B: Overview of the Akiyoshi Limestone and the Tsunemori Formation (lower and upper part). C: Less deformed mudstone of the Tsunemori Formation including thin layers of calcarenite (white part). D: Lower part of the outcrop (Fig. 4B), where mudstone of Tsunemori Formation are tectonically intercalated within the Akiyoshi Limestone. Mudstone with limestone clasts injected into the Akiyoshi Limestone.

**Fig. 5:**
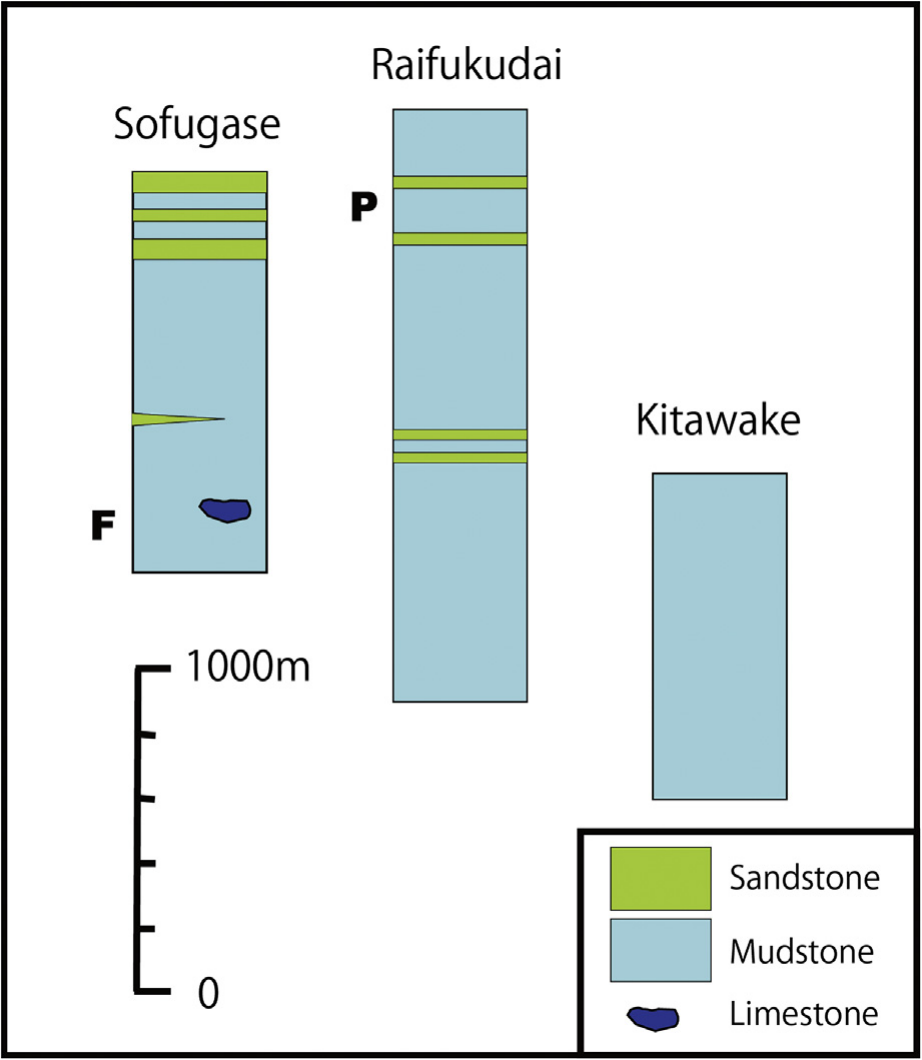
Stratigraphic columns of Tsunemori Formation in three areas, which are divided by faults. “P” and “F” show the stratigraphic position of pebbly mudstone and fossil occurrence of *Follicucullus* cf. *schorasticus.*

Tsunemori Formation is distributed in the western part of Mine City (Fig. 2). It is composed mainly of mudstone or mudstone dominant turbidites intercalated with minor amount of sandstone dominant turbidite (Fig. 3). It is locally associated with pebbly mudstone, calcarenite and limestone breccia. The Tsunemori Formation is contact with low angle thrust faults (Figs. 2 and 3). However, the Tunemori Formation lies on the Akiyoshi Limestone with irregular boundaries at the Sumitomo Cement Shuho Mine (Fig. 4A, B). And the mudstone of Tsunemori Formation is injected into the Akiyoshi Limestone with angular fragments of the Akiyoshi Limestone (Fig. 4D). Although their original relationship is discussed variously, there is no clear evidence on it (e.g. Fujii and Mikami, 1970; Sano and Kanmera, 1991d). The Tsunemori Formation is unconformably overlain by Triassic shallow marine to non marine sedimentary rocks of the Mine Group, which is widely distributed in the west of Mine City (Kametaka, 1999). The Tsunemori Formation is divided into three blocks by vertical faults. The stratigraphic columns of these three tectonic blocks are shown in Fig. 5.

**Fig. 6:**
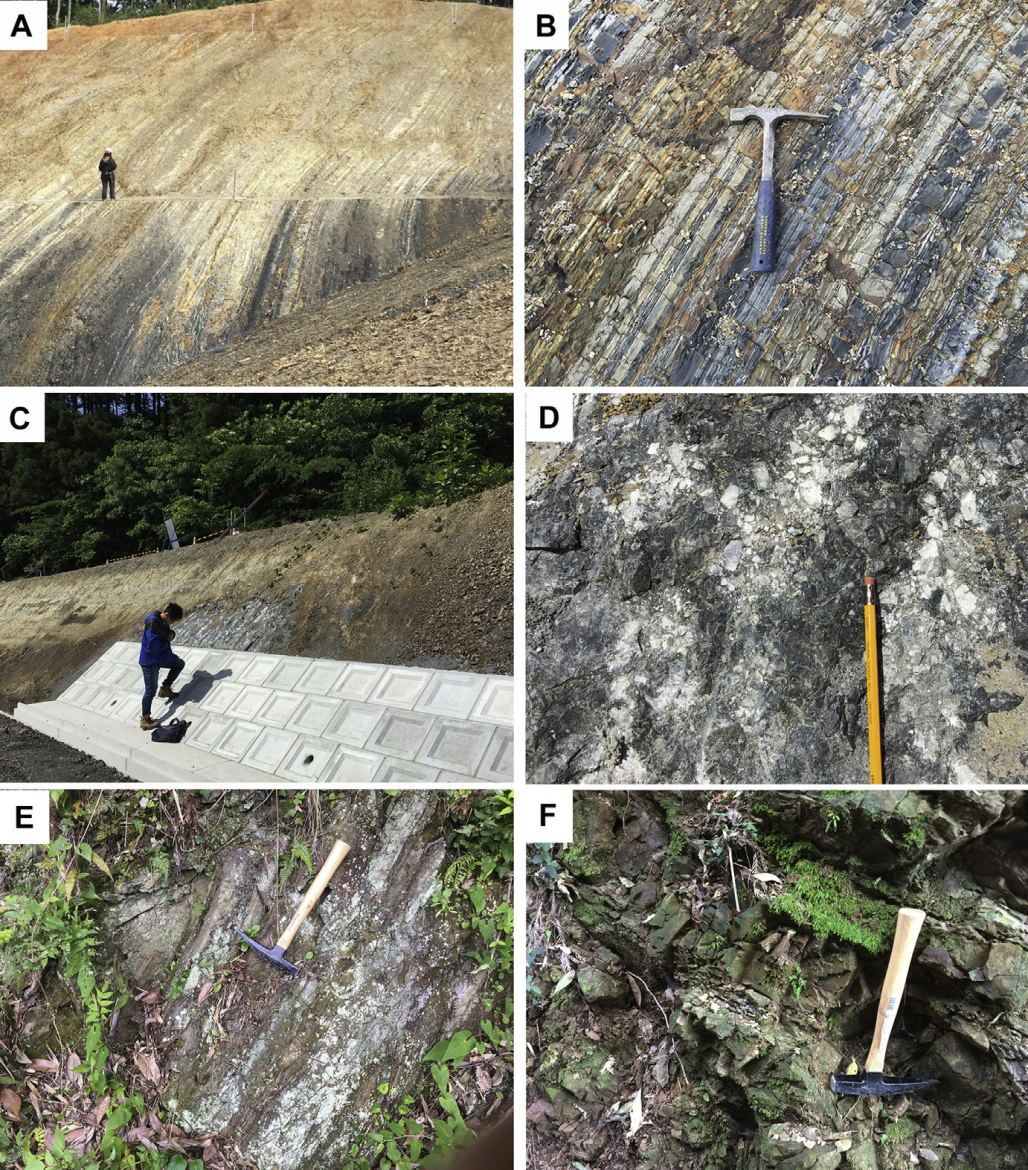
Typical lithology of Tsunemori Formation. The locality of Fig. 6A–D is shown as “F” in Fig. 3, while the localities of Fig. 6E–F is shown as “T” in Fig. 3. A: Overview of the outcrop of mudstone dominant turbidite, Sobugase, Mine City. B: Close-up of the outcrop of mudstone dominant turbidite (right hand site of Fig. 6A). C: Occurrence of limestone breccia within the Limestone breccia intercalated within the mudstone- dominant turbidite as a block of 3 m in diameter. D: Close-up of the outcrop of limestone breccia. E: Mudstone dominant turbiite at Tsunemori, Mine City. F: Alternation of sandstone and mudstone at Tsunemori, Mine City.

**Fig. 7:**
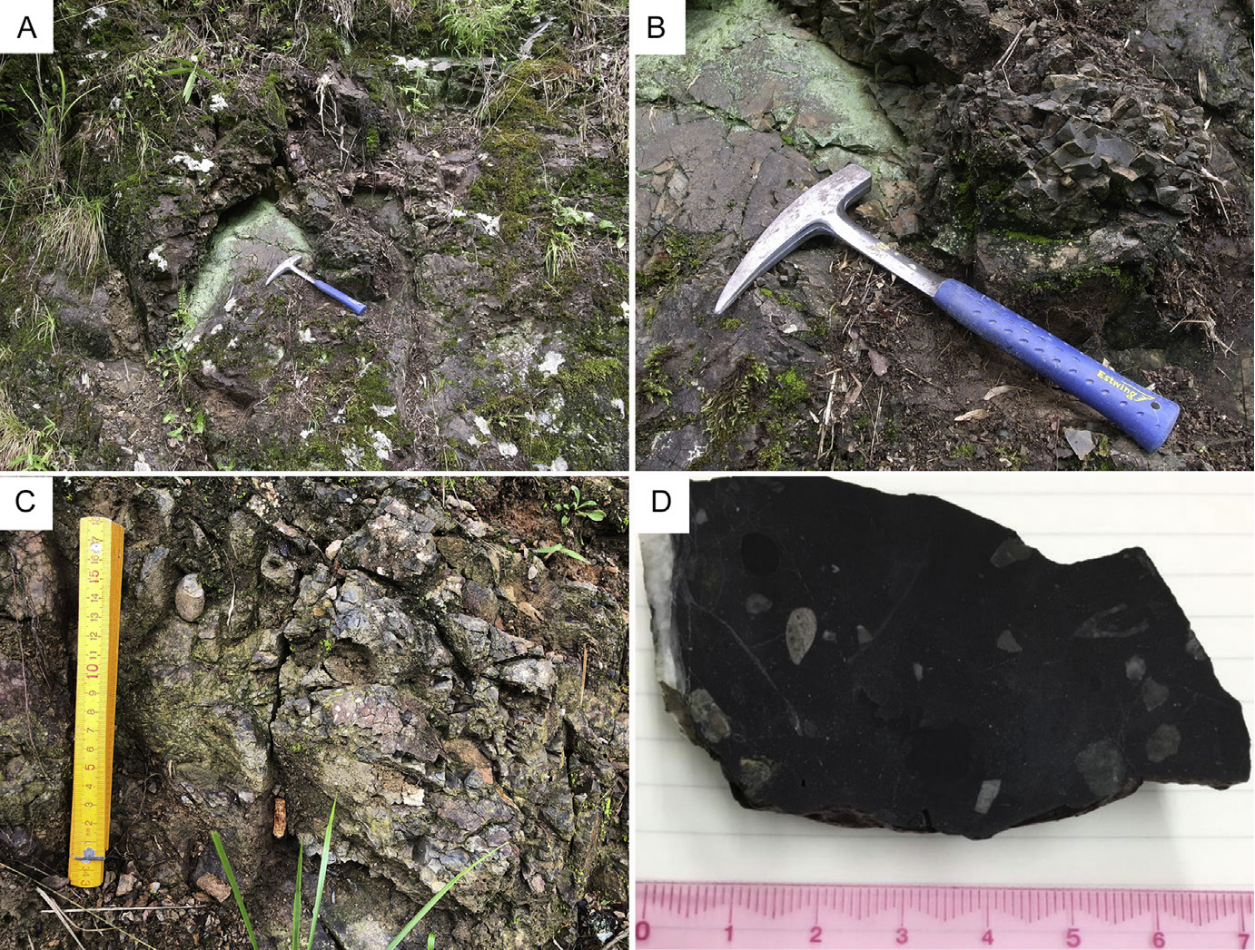
Outcrop of pebbly mudstone and polished hand specimen. The locality is shown as “P” in Fig. 3. A: Outcrop of pebbly mudstone of Tsunemori Formation. B: Close-up of Fig. 7-A showing smooth texture without any fissility and shear planes. C: Pebbly mudstone including rounded pebbles. D: Polished surface of hand specimen of pebbley mudstone of this outcrop.

Mudstone and mudstone dominant turbidite is widely distributed in the Tsunemori Formation. Mudstone beds are interbedded with very thin layers of sandstone or siltstone. The muddy turbidite sequences are well stratified without layer parallel faults, although they are gently folded. It is locally intercalated with limestone breccia (Fig. 6D) and layers of calcarenite (Fig. 4C). Limestone breccia is intercalated as blocks in alternation of sandstone and mudstone. Conglomerate is more than 10m in thickness, and contains pebbles of felsic to intermediate igneous rocks, limestone and sandstone. Calcarenite is composed of limestone fragments and forms thin beds in mudstone (Fig. 4C). Pebbly mudstone is a few meters in thickness, and contains limestone, chert and felsic igneous rocks within mudstone matrix. The mudstone and mudstone matrix of pebbly mudstone yield no features of tectonic shearing such as fissility and scaly cleavages (Fig. 7).

### 2.2 Akiyoshi Limestone

The Akiyoshi Limestone is distributed in the Mine City in the central part of Yamaguchi Prefecture, Japan (Fig. 2). It is composed mainly of white pure limestone including various fossils ranging in age from late Early Carboniferous to late Guadalupian. It is divided into four facies such as skeletal - oolitic grainstone, muddy limestone, muddy limestone - skeletal grainstone, and reefal limestone (Sano, 2006).

The limestone is underlain by basalt, which erupted to form an “off-ridge seamount” during Carboniferous time. The paleomagnetic data revealed that the seamount on which Akiyoshi Limestone was build up was formed at the low latitude area of the Panthalassan Ocean (Fujiwara, 1967). It includes various shallow marine fossils such as fusulinoideans, corals, brachiopods, crinoids and calcareous algae. Ota (1977) divided the Akiyoshi Limestone into ten fusulinoidean zones, i.e. *Fusulinella bicnica, Triticites simplex, Pseudofusulina vulgaris, Pseudofusulina ambigua, Misellina claudia, Parafusulina kaerimizuensis, Afghanella schencki, Neoschwagerina craticulifera, Verbeekina verbeeki* and *Colania douvillei* Zones. The Akiyoshi Limestone ranges in age from late Visean to Capitanian.

Permian accretionary complex of the Akiyoshi Belt is widely distributed in this area. The Akiyoshi Limestone, a massive white to light gray limestone, is distributed in the central part (Fig. 2). Tsunemori Formation occurs in the western part of this area. Oda unit is distributed on the southeast of Akiyoshi Limestone are occupied by Oda unit, while the northwest area is underlain by Beppu unit (Fig. 2). The Oda and Beppu units are composed mainly of pelagic chert, hemipelagic siliceous mudstone, alternation of sandstone and mudstone, massive sandstone and conglomerate, chert breccia in ascending order.

### 2.3 Sedimentation age of Tsunemori Formation

**Fig. 8:**
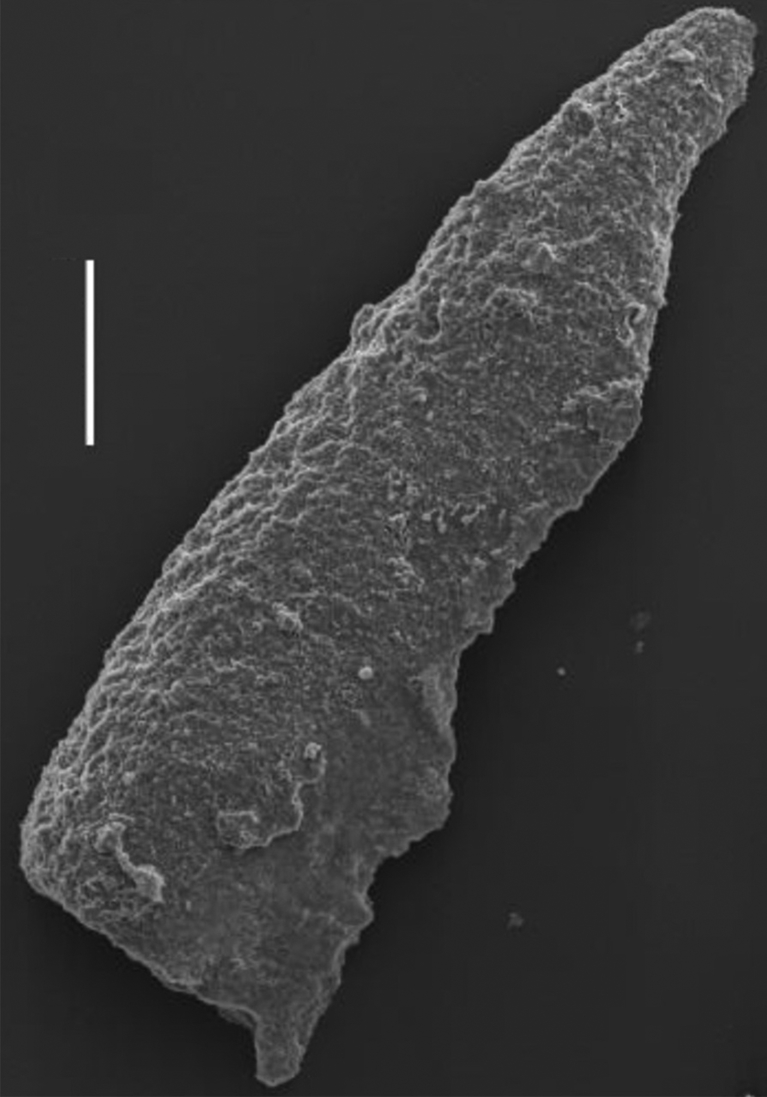
Late Middle to early Late Permian radiolarian: *Follicucullus* cf. *schorasticus* Ormiston and Babcock. The fossil was extracted from mudstone in the turbidite at the locality “F” in Fig. 3. White bar shows 100μm.

Early Late Permian radiolarians including *Follicucullus scholasticus* Ormiston and Babcock (Fig. 8). It was extracted from mudstone part of mudstone dominant turbidite, which is exposed in Sobugase area, Mine City at the point “F” in Fig. 3. It is stratigraphically lower part of the Tsunemori Formation shown as “F” in Fig. 4. The mudstone sample was crushed into small fragments, and put in plastic beaker with ca. 5% hydrofluoric acid for about 24 hours. Residue was collected on the floss of 37 μm, and was boiled with c. 10% hydrochloric acid. After washing the residue, radiolarians were picked up under the microscope. The fossils are observed and photographed in the Scanning Electron Microscope of Yamaguchi University. *Follicucullus* cf. *scholasticus* Ormiston and Babcock is identified (Fig. 8), together with fragments of sponge spicule.

Various types of fossils such as foraminifera, fusulinoideans, brachiopods and radiolarians in mudstone were reported in the previous works. Sano and Kanmera (1991a) found *Pseudoalbaillella* sp. aff. *P. longicornis* Ishiga and Imoto, *Follicucullus monacanthus* Ishiga and Imoto and *Follicucullus scholasticus* m. I. Ishiga from scaly mudstone. Kanmera et al. (1990) reported Permian radiolarians, P*seudoalbaillella fusiformis* (Holdsworth and Jones), *Pseudoalbaillella* sp. aff. *P. longicornis*, *Follicucullus monacanthus* Ishiga and Imoto, and *Follicucullus scholasticus* Ormiston and Babcock from mudstone and acidic tuff in the west of Akiyoshi. These assemblage indicates *Follicucullus monacanthus* Zone *and* Zone of late Middle Permian to earliest Late Permian. The occurrence of *Follicucullus* cf. *scholasticus* in the turbidite of Tsunemori Formation at Sobugase area in this study supported the age determination of previous works.

Some other fossils of older age than radiolarians are reported in the Tsunemori Formation. Middle Permian fusulinoideans including *Lepidolina multiseptata shirakawaensis* (Ozawa) and Neoschwagerinidae gen. et sp. indet were reported by some authors (i.e. Fujii and Mikami, 1970; Tazawa et al., 2009). Yanagida (1996) and Tazawa et al. (2009) found various types of brachiopods in the Tsunemori Formation, such as *Chonetinella krotovi* (Fredericks), *Megousia* sp., *Eolyttonia* sp. and *Spiriferella persaranae* Grabau. These fossils are considered to be reworked fossils (Tazawa et al., 2009). However, Tazawa et al. (2009) also reported that some of brachiopods, fusulinoideans and crinoids of late Late Permian age occurred within the mudstone matrix of the pebbly mudstone of the Tsunemori Formation.

## 3 Discussion

### 3.1 How are seamount fragments incorporate into accretionary wedge?

Akiyoshi Limestone is atoll carbonates developed on volcanic seamounts in Late Paleozoic ocean (Sano and Kanmera, 1988; Sano et al., 2004; Sano, 2006). Sano and Kanmera (1991d) proposed atoll carbonate collapse model to explain the relationship between Akiyoshi Limestone and Tsunemori Formation. However, it is difficult for atoll carbonates to accrete within an accretionary prism at trench, if we take into account the present condition of convergent margin. Thousands of seamounts are distributed in present Pacific Ocean and Philippines Sea (Wessel, 2001). However, these seamounts cannot incorporate into accretionary prism easily. In the case of tectonic erosion boundary like Japan Trench, seamounts were collapsed at the trench, but subducting slab brings the seamount fragments into deep mantle (Von Huene and Culotta, 1989). On the other hand, at Nankai Trough, a typical accretionary boundary, seamounts do not break up to cause seamount collapse at the trench, and subducted into accretionary wedge of soft sediments without any deformation at the Nankai Trough (Yamazaki and Okamura, 1989; Okamura, 1991). Therefore, it is unlikely for seamount accretion to occur at the trench or the shallower part of accretionary wedges.

Taking into account the present convergent margin, the atoll carbonates on seamounts is difficult to be accreted at trench. Where is the possible location for limestone to be detached from seamount and to be accreted within the wedge? Wakita (1998, 2000, 2012) suggested that decollement gradually steps down into the deeper part of the accretionary wedge, and that limestone on subducting seamount must be detached by underplating in the deep portion of the accretionary wedge.

### 3.2 Meaning of reworked fossils, calcarenite, limestone breccia, and undeformed mudstone

Sano and Kanmera (1991a, b, c, d) investigated the contact between Akiyoshi Limestone and Tsunemori formation at the Yamaguchi quarry of Shuho Mine, Sumitomo Osaka Cement Co. Ltd. and Amagoiyama quarry of the Isa Cement Factory, Ube Inddustries Ltd. Sano and Kanmera (1991a) described scaly cleavages on mudstone with limestone blocks, and explained it as evidence of melange formation caused during subduction and accretion. However, scaly cleavage on mudstone of Tsunemori Formation is developed only in the area for the Akiyoshi Limestone to distribute widely (blue color part in the central to northeastern area of Fig. 2). In the area, the Akiyoshi Limestone tectonically associated with minor amount of the Tsunemori Formation. In other area far from the Akiyoshi Limestone (Study area in Fig. 2), both of mudstone and sandstone of the Tsunemori Formation is not severely sheared (Figs. 6 and 7).

Sedimentary rocks forming accretionary complex was deformed in various degrees during accretionary processes. Some of them were completely deformed to form mélanges, and the others form broken formation or tectonically stacked turbidite. The turbidite is sometimes cut by faults parallel or subparallel to the bedding, and some of sandstone beds of the turbidite show pinch and swell structures in ancient accretionary complexes (e.g., Wakita, 1988, 2000). However, sandstone and mudstone beds of the Tsunemori Formation are much less deformed than the ones of ancient accretionary complexes. These are well stratified without pinch and swell structures and layer parallel faulting (Fig. 6A, B). No fissility and scaly cleavage are developed in mudstone and pebbly mudstone of Tsunemori Formation (Figs. 6 and 7). These sedimentary and deformation structures suggest that the Tunemori Formation was not formed by successive accretion of ocean plate stratigraphy at the trench.

Tsunemori Formation is locally intercalated with beds of calcarenite and blocks of limestone breccia. Calcarenite composed of calcite or limestone fragments within finer fragments of calcium carbonate minerals, without detrital fragments like quartz and feldspars. The calcarenite beds are usually 5–20 cm in thickness, and are intercalated within mudstone (Fig. 4C). Limestone breccia is dominant along the margin of Akiyoshi Limestone as Sano and Kanmera (1991a, b, c, d) described. Limestone breccia was deposited in proximal, while calcarenite was deposited in distal part from the source. As calcarenite was composed mainly of limestone fragments without detrital fragments, the source must be composed of pure limestone without other rock types. The presence of calcarenite without detrital fragments suggests that the source of carbonate fragments must be different from the mudstone and sandstone of the Tsunemori Formation.

Tsunemori Formation yields various types of fossils such as fusulinoideans and brachiopods. Fusulinoideans and brachiopods in the Tsunemori Formation are considered to be reworked fossils (Kanmera et al., 1990; Yanagida, 1996; Tazawa et al., 2009). If these fossils are derived fossils, it is necessary for Akiyoshi Limestone to be weathered and eroded prior to the formation of these fossils. In the atoll carbonate collapse model as proposed by Sano and Kanmera (1991a, b, c, d), it is difficult for the fossils to erode out from the limestone and entered in the mudstone matrix of the pebble mudstone as Tazawa et al. (2009) described. The deep ocean trench expected as the site for the Tsunemori Formation is not suitable for weathering and erosion of the Akiyoshi Limestone. Tazawa et al. (2009) mentioned that some of brachiopods, such as *Lanimargus japonicas*, *Megousia* sp. and *Eolyttonia* sp. are not derived fossils, and indicates the age of the upper part of Tsunemori Formation is late Late Permian. These fossils are very close in the age of sedimentation shown by radiolarians from turbidite and detrital zircon from turbidite sandstone. It may have been able for calcareous fossils to form a reef on the exposed limestone at the arc trench gap. In the same way, Arita et al. (2001) interpreted the limestone of Kozaki Formation of Kurosegawa Tectonic Belt as autochthonous based on their observation and the description of Kanmera (1961).

### 3.3 New tectonic model for subduction and accretion of the Akiyoshi Limestone

If atoll carbonate collapses at tectonic erosion margin, the product cannot accrete. On the other hand, atoll carbonate cannot collapse at the accretionay margin. Therefore, trench is not suitable place for atoll carbonate to collapse and accrete to the accteionary wedges. If so, where is the possible site for the atoll carbonate collapse, accretion and mixing with detrital sediments? One possibility is the tectonic mixing within the accretionary wedges as is shown by Wakita (2012). However, the deformation style of the Tsunemori Formation is very different from one of the accteionary complex of Mino-Tamba-Chichibu Belt formed by tectonic mixture during oceanic plate subdution (Wakita, 2012).

The sedimentary sequences of the Tsunemori Formation are well stratified without pinch and swell structures and layer parallel faulting. No fissility and scaly cleavage are developed in mudstone and pebbly mudstone of the Tsunemori Formation. Atoll carbonate collapse at the trench cannot give the time and place to erode limestone to provide reworked fossls into Tsunemori Formation. Presence of calcarenite and limestone breccia suggest that limestone is collapse, eroded and provided their fragments into the muddy deposits of the Tsunemori Formation.

**Fig. 9:**
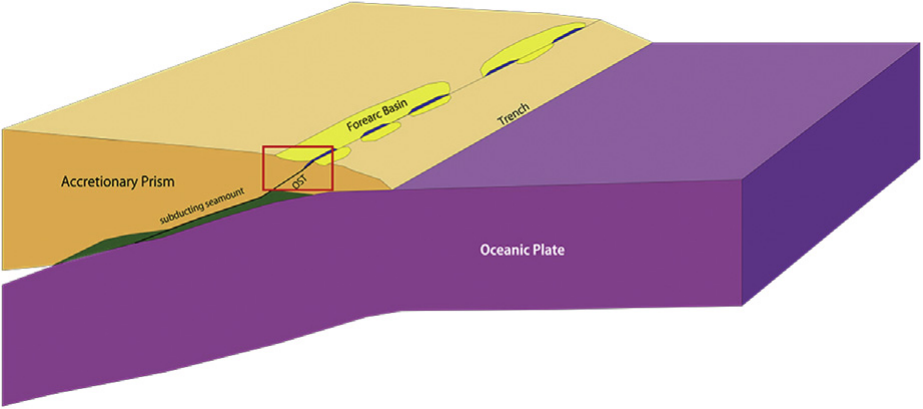
Permian subduction and accretion system for the Akiyoshi Belt. Atoll carbonates were truncated by *decollement*, and were accreted into the deep part of the accretionary wedge in Late Permian. After the detachment, the accreted carbonates moved upward to reach arc trench gap. (OST: out-of-sequence thrust).

**Fig. 10:**
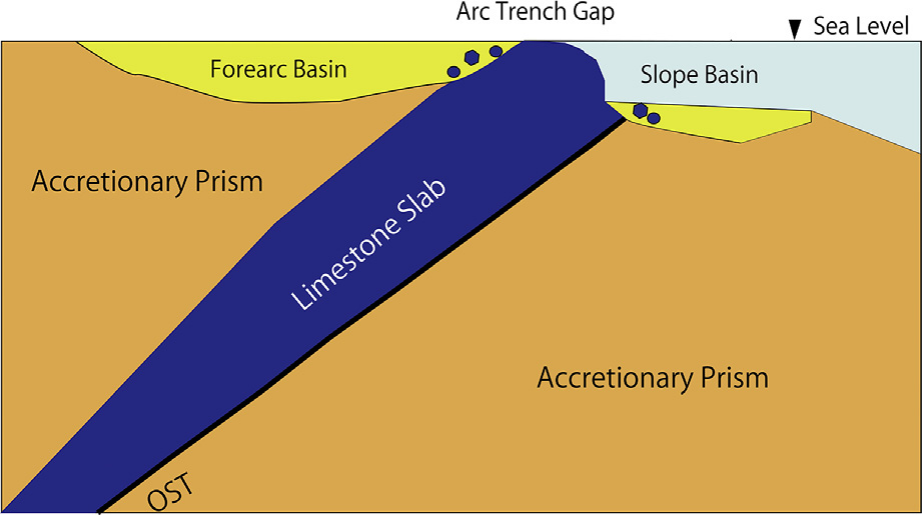
New tectonic model for the Tsunemori Formation and the Akiyoshi Limestone At the arc trench gap, the Akiyoshi Limestone provided limestone fragments into forearc basin or slope basin on the Permian accretioanry wedge of the Akiyoshi Belt.

Based on the observation on sedimentary and deformation structures of Tsunemori Formation, sedimentary rocks of Tsunemori Formation are different in deformation style from sedimentary formation deposited at trench and accreted to form accretionary wedge. Present authors proposed the forearc basin or trench slope basin as sedimentation site for the Tsunemori Formation instead of trench proposed by Sano and Kanmera (1991a, b, c, d). Accreted limestone in the deeper part of accretionary wedge must rise upward along out-of-sequence thrust and may reach at arc trench gap (Figs. 9 and 10). In the event that the sea level was low and accretionary wedge developed high, arc trench gap may appear on the sea. The limestone was weathered and eroded to provide derived fossils, and was collapsed into forearc and/or slope basins because of upward movement of accreted limestone blocks along splay fault or out-of-sequence thrust (OST) (Fig. 9). As Tazawa et al. (2009) suggests some of brachiopods lived during the deposition of Tsunemori Formation, such fauna may be able to live on arc trench gap, and entered into the forarc basin.

The Akiyoshi Limestone is detached from subducting seamount and accreted at the deep portion of the accretionary wedge. It moved upward along out-of-sequence thrust to reach arc trench gap. The Akiyoshi Limestone was weathered and eroded to provide derived fossils, and collapse into forearc and/or slope basin to provide calcarenite and limestone breccia. Newly proposed model suggests that Tsunemori Formation is a deposit of forearc basin and/or slope basin rather than trench deposits. A part of the Akiyoshi Limestone cropped out at the arc trench gap and provide limestone fragments and blocks into the sedimentary basins where the Tsunemori Formation was deposited.

Although this paper disproves the previous theory proposed by Sano and Kanmera (1991d) on atoll carbonates collapse at the trench for the Akiyoshi Limestone, there is one possibility to accept their theory. If ocean plate subduction stopped just after atoll carbonate collapse at the trench, limestone breccia derived from the collapsed atoll carbonate can be preserved in the trench sediments. The trench sediments must have escaped from the tectonic deformation caused by successive plate subduction. We need to consider this possibility to understand how the Tsunemori Formation was formed in future.

Recumbent fold and overturned structure of Akiyoshi Limestone is well known form the early stage of research in the Akiyoshi area (Ota, 1977). Sano and Kanmera (1991d) proposed that the gradual collapse of Akiyoshi Limestone could produce the overturned stratigraphy in the Akiyoshi Limestone. However, we can propose an alternative solution of the overturned limestone occurrence. Kimura and Hori (1993) shows that the lower parts of accreted OPS are sometimes overturned to form a duplex during accretionary processes. Thrust movement along the OST may have caused the tight folding of Akiyoshi Limestone. Present example of the arc trench gap exposed above sea level can be observed in the forearc region along the Sunda Trench (Moore et al., 1982).

## 4 Conclusions

We proposed forearc basin or trench slope basin for the depositional site instead of trench in this paper. The sedimentary sequences of the Tsunemori Formation are well stratified without pinch and swell structures and layer parallel faulting. No fissility and scaly cleavage are developed in mudstone and pebbly mudstone of the Tsunemori Formation. These styles of deformation suggest that the Tsunemori Formation was not a part of accretionary wedges, but possibly was deposits in fore-arc basin or slope basin. A part of the Akiyoshi Limestone cropped out at the arc trench gap and provided calcarenite and limestone breccia into the Tsunemori Formation. Reworked fossils derived form the limestone and some of brachiopods living in late Late Permian were derived from the arc trench gap into the forearc basin or slope basin formed on the pre-exist accretionary complex.

## Declarations

### Author contribution statement

Koji Wakita: Conceived and designed the experiments; Performed the experiments; Analyzed and interpreted the data; Contributed reagents, materials, analysis tools or data; Wrote the paper.

Ruri Yoshida, and Yuki Fushimi: Performed the experiments.

### Funding statement

This work was supported by JSPS KAKENHI Grant Number JP16K05579.

### Competing interest statement

The authors declare no conflict of interest.

### Additional information

No additional information is available for this paper.
